# Neglected cutaneous skin malignancy: A patient with concurrent giant basal cell carcinoma and melanoma

**DOI:** 10.1002/ski2.68

**Published:** 2021-10-05

**Authors:** L. Sun, E. Tan

**Affiliations:** ^1^ Department of Plastic Surgery Waikato Hospital Hamilton New Zealand

## Abstract

**Background:**

Giant basal cell carcinomas (BCCs) are a rare subtype of BCC that grow to be greater than 5 cm in diameter. With the increase in size, there is a corresponding increase in metastatic rate and state of local invasion, with a clinical morphology that can be hard to differentiate from other subtypes of cutaneous malignancy. Although histologically equivalent to their common sub‐centimetre counterparts, giant BCCs can precipitate significant systematic medical morbidity as well as psychological trauma, and can be a real surgical reconstructive challenge to clinicians.

**Aims:**

To add breadth to the existing cases in the literature, as well as a fresh patient perspective on the psychological challenges in a patient with Giant BCC.

**Materials & Methods:**

A case from the Waikato Hospital, New Zealand referred to the Plastic and Reconstructive Department is carefully photographed, ordered, and presented.

**Results:**

We present a case of a 15 cm giant BCC of the back existing alongside a neglected thick exophytic melanoma of the elbow in a patient who had been too embarrassed to approach healthcare professionals. These skin lesions were an incidental discovery by the general practitioner after the patient presented with symptoms of shortness of breath.

**Discussion:**

Neglected skin cancers can fungate and be clinically morphologically confusing. Photographs of examples of these tumours can hone clinician awareness of the existance of Giant BCCs.

**Conclusion:**

Giant BCCs are an entity yet to receive standardized treatment stratification. Prompt diagnosis and staging scans mean an expedited path to wide local excision and reconstruction, resulting in timely resolution of patients' immediate morbidity from their oncological disease burden.

## Graphical abstract

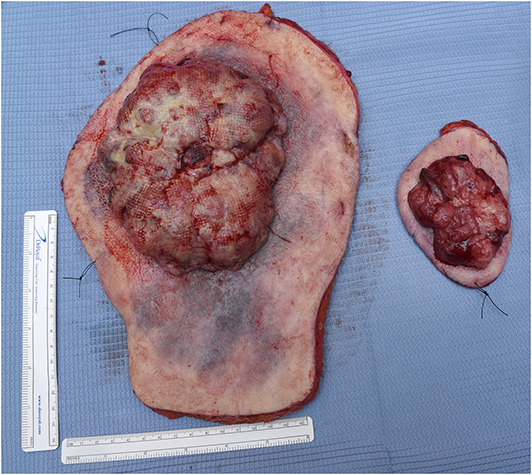

1

**What’s already known about this topic?**

- Giant Basal Cell Carcinomas (BCCs) larger than 5 cm in diameter have far greater metastatic potential and can be a source of patient embarrassment as well as a reconstructive challenge after surgical excision. Due to their rarity, optimal management in terms of oncological margins and adjuvant therapy have yet to be standardized.

**What does this study add?**

- Giant BCCs can appear macroscopically indistinguishable from other ulcerated malignancies and their systemic complications are far‐reaching. BCCs, a typically low risk skin cancer, can become costly to both patients and healthcare systems through serial neglect. Clinicians should note that, from a patient perspective, oncological clearance can be of secondary importance to symptom control and comfort.

## 2 INTRODUCTION

The incidence of basal cell carcinoma (BCC) was estimated as 1177 per 100,000 in the Auckland region,^1^ and they are by far the most common cutaneous malignancy worldwide. Giant BCC (GBCC –defined as BCCs with a greater than 5 cm diameter) are rare: a recent 2019 UK study in a tertiary oncology centre saw only 43 patients with GBCC in a 20 year period.^2^ To the best of our knowledge, there are currently no studies on GBCC incidence in New Zealand. It is still unclear whether GBCCs differ significantly morphologically or genetically from regular BCCs or whether their large size and rapid growth is the natural history of a regular BCC in patients who have not received timely treatment. GBCC local recurrence or metastasis rate is 38.3%,^3^ which contrasts greatly with the indolent nature of BCCs. There are only approximately 350 cases to date^4^ of metastatic BCC, with incidences reported in a variety of anatomical locations.^5^ Melanomas are a far better‐studied entity than GBCCs, with reliably matched decreases in expected 10 years survival rates with increasing disease stage.^6^

## 3 CASE REPORT

**FIGURE 1:**
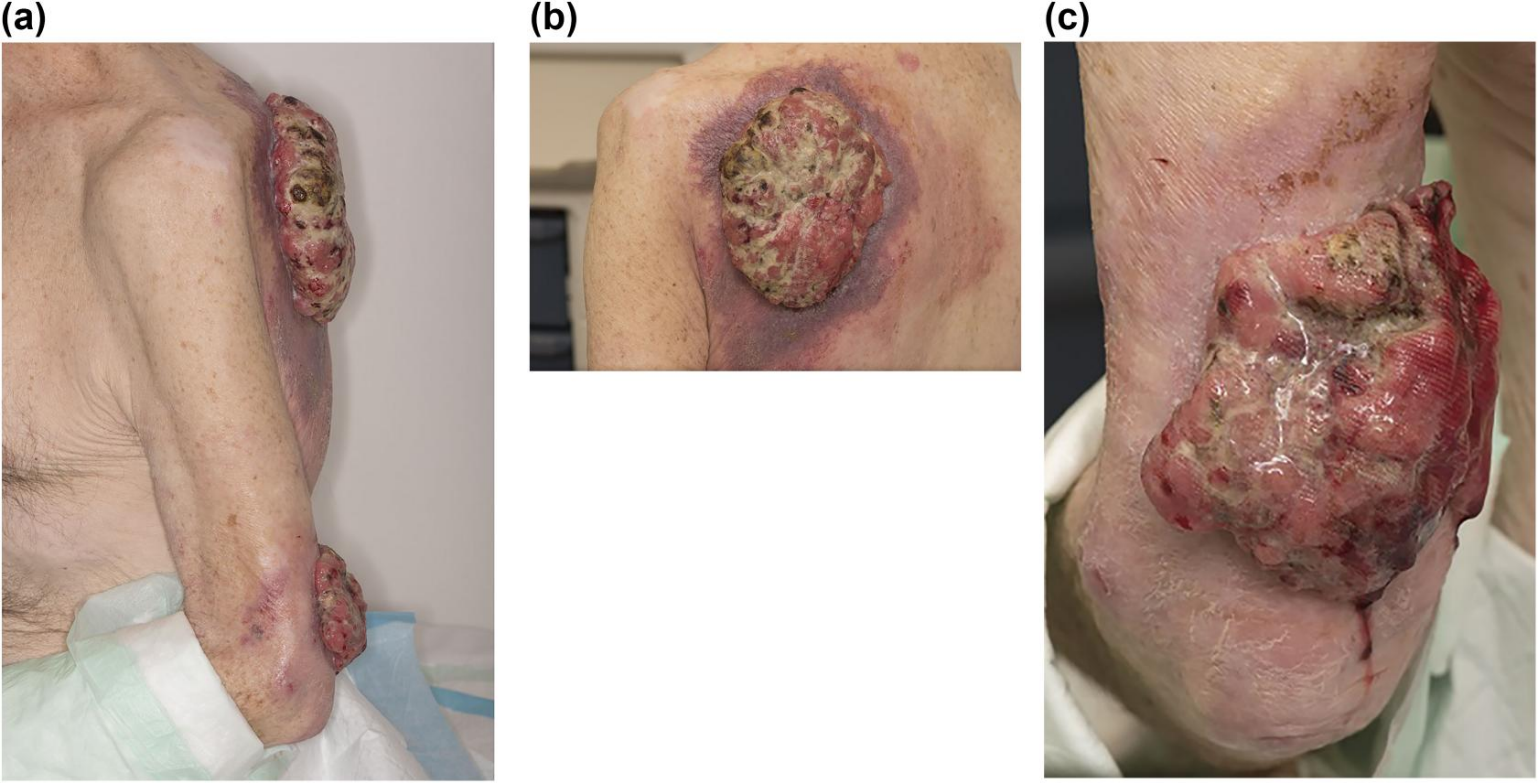
(a) GBCC on patient's back with ulcerated melanoma on posterior elbow. (b) GBCC close‐up. (c) Melanoma of elbow close‐up. Note the similarities in appearance between (b and c) macroscopically, it can be hard to tell large, mature, ulcerated lesions apart. Pseudomonoal colonization further complicates lesion morphology. Patient cachexia can be appreciated

Our patient is a 74‐year‐old Caucasian gentleman presenting with malodorous ulcerated and exophytic lesions over his left back (15 cm in diameter) and elbow (6 cm) that have steadily grown over the last 2 years (Figure 1). He had no current co‐morbidities and was an ex‐smoker. His lesions were an incidental finding after presenting to his general practitioner for new onset shortness of breath on exertion.

He stated that the delay in contacting a healthcare professional about the lesions was due to embarrassment and worries about judgement he may receive from a doctor for leaving his lesions for this long. A further stigmatizing element of the lesion was the ulcerated and anaerobic surface promoting offensive‐smelling *pseudomonas* biofilm colonization, resulting in increased embarrassment hindering presentation to medical services, as well as a challenge for the patient in terms of social interaction. Living alone, he had a severely limited support network; his only family was a sister with whom he was just in telephone contact.

The patient was referred to the acute service but was fast‐tracked into a consultant clinic and underwent point of care incisional biopsies to rapidly obtain histological diagnosis. He had a severe microcytic iron deficiency anaemia (haemoglobin of 66 g/dL) which was attributed to persistent oozing from the friable surface of his back lesion. This is a common finding in up to 35% of presenting patients with GBCC.^3^ Subsequent hospital admission and packed red cell and iron transfusions resolved his shortness of breath acutely. An urgent inpatient staging whole body CT revealed that, surprisingly, there were no solid organ or bony metastases. A few lymph nodes were however visualized in the axilla and the subpectoral region on the ipsilateral side (later biopsied under ultrasound revealing them to be reactive only). Follow‐up MRIs of the lesions confirmed the absence of local invasion and intact fat and fascial planes between the tumour and deeper structures.

**FIGURE 2:**
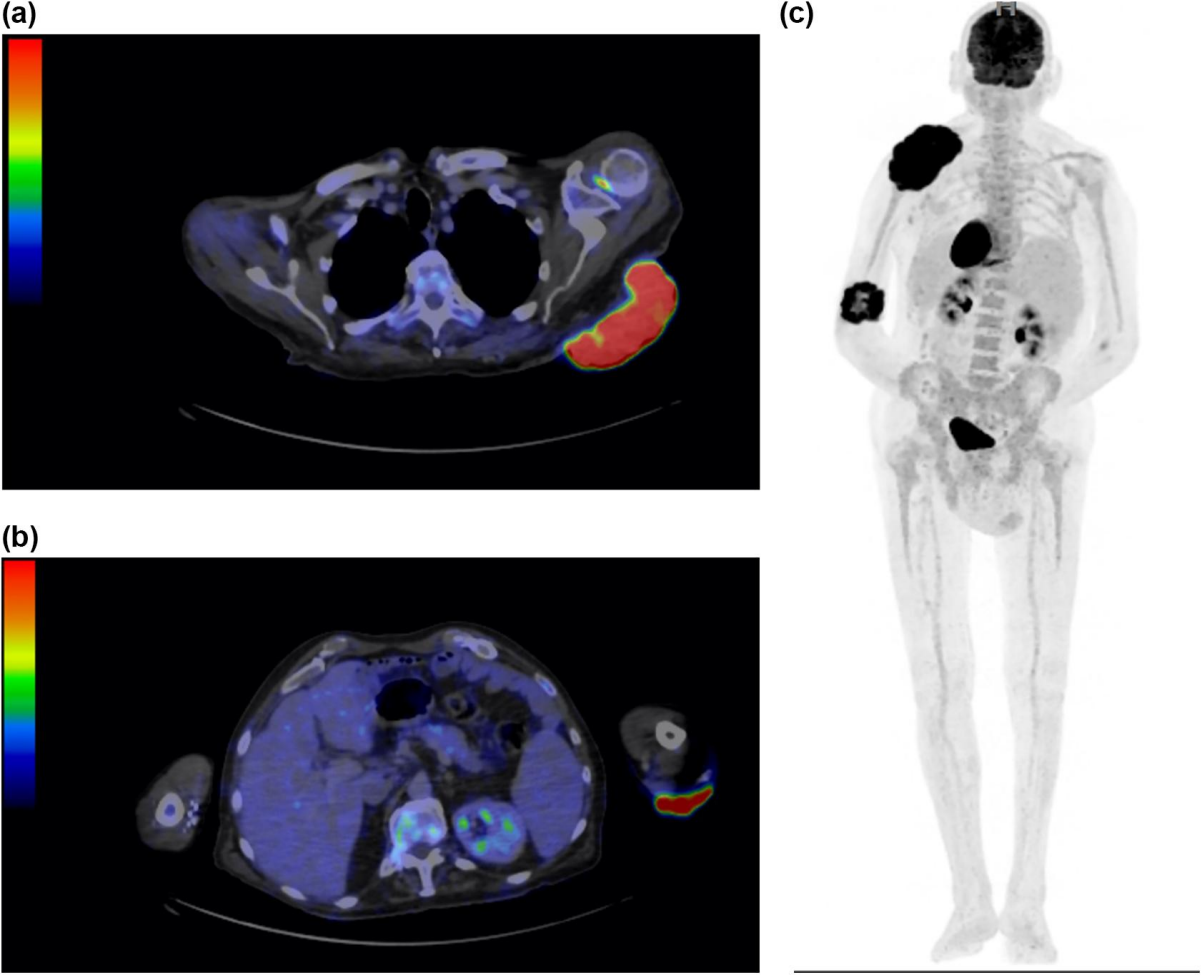
(a) Axial section of PET‐CT scan showing FDG avidity in the GBCC left scapula, as well as FDG‐avid node in axilla (later found to be benign on biopsy). (b) FDG avidity in left elbow indicating melanoma. (c) The absence of metastatic‐looking large distant deposits on PET‐CT

Immunohistochemistry of biopsies taken showed SOX10 and Melan‐A positivity for the elbow mass and CK positivity for the back lesions, representing a histological diagnosis of malignant melanoma (20 mm Breslow thickness, nodular subtype, dermal mitoses of 2/mm^2^) for the former and BCC for the latter. An urgent PET‐CT scan confirmed no obvious metastatic regions of FDG‐avidity (Figure 2). After a discussion with the melanoma multi‐disciplinary meeting, it was decided that surgical resection with curative intent should be the first option for treatment for both lesions. Biopsy of sentinel node was discussed with patient, but active surveillance of axillary lymph node basin was chosen instead due to patient preference.

**FIGURE 3:**
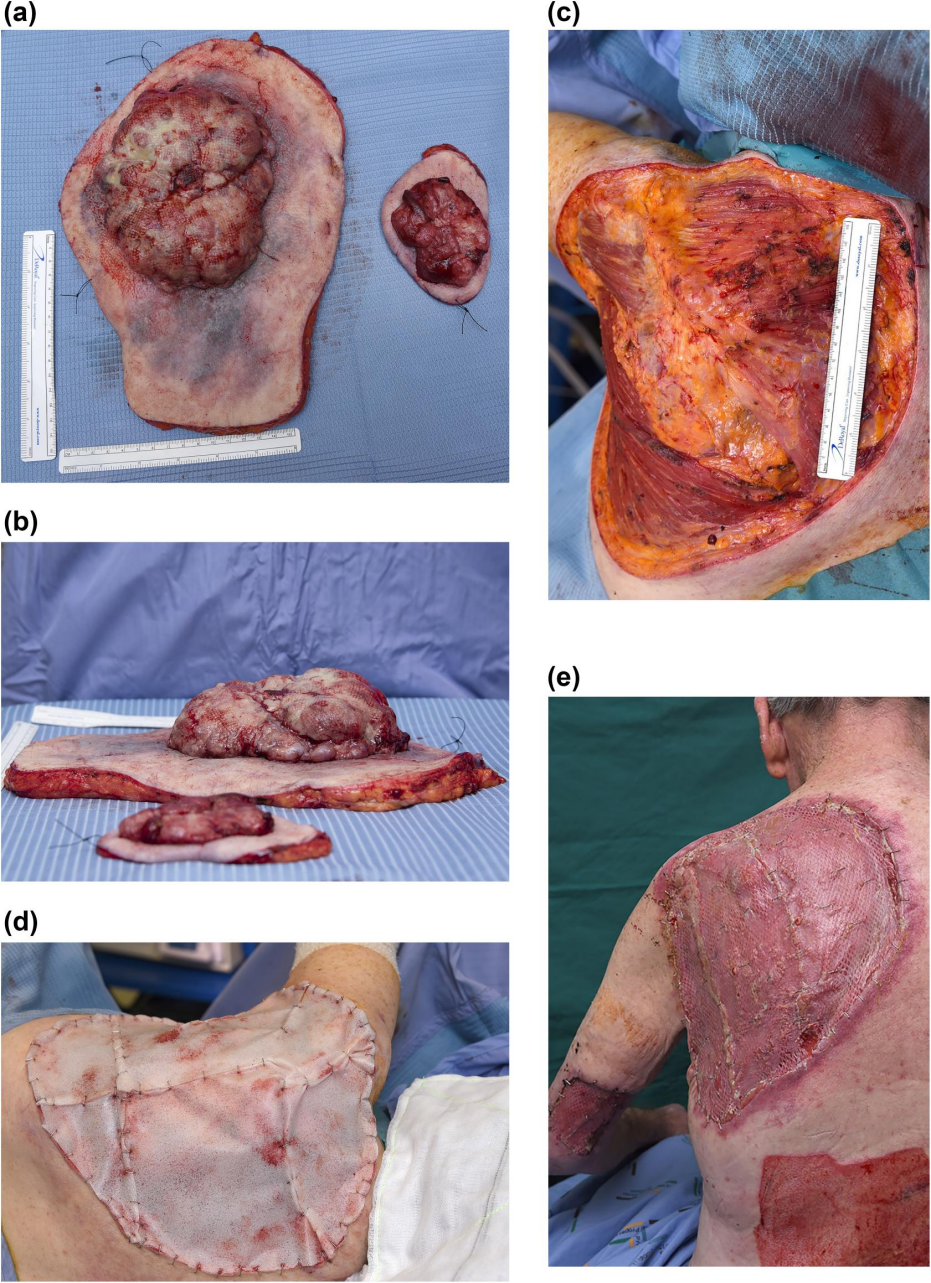
(a) Lesions were excised with a 2 cm margin around the area of skin change. (b) resection specimens – GBCC (larger) and melanoma (smaller) laid side by side. (c) Large post‐resection tissue defect with exposed trapezius and latissimus dorsi muscles. (d) Biodegradable temporizing matrix (BTM) used to cover defect, which was stapled in place. (e) This BTM was later delaminated at 4 weeks, and the granulation tissue was skin grafted with the donor site of the lower back

Excision and tissue reconstruction proved to be more complex than the majority of BCC or melanoma excisions (which are often directly closable or require a small skin graft). There currently is no widely accepted guidance on surgical resection margin in patients with GBCC due to a paucity in cases in the literature, and furthermore, the dimensions of the lesion in this case report come close to classification criteria for a supergiant BCC (>20 cm in diameter). On the day of definitive surgery, 34 days after GP referral, it was decided that the circumferential reactive‐looking skin change around the lesion itself could represent microscopic local invasion, and a further 1 cm diameter was marked around the skin change as our excision margin. This resulted in a significant soft tissue defect down to muscle (almost 30 cm × 30 cm), and, due to the back being a dependent area which needs to stand up to regular shearing forces (clothing) and pressure (when lying down to sleep), biodegradable temporizing matrix (BTM) was chosen to provide a thicker layer of granulation tissue to skin graft onto as a second staged procedure (Figure 3).

**FIGURE 4:**
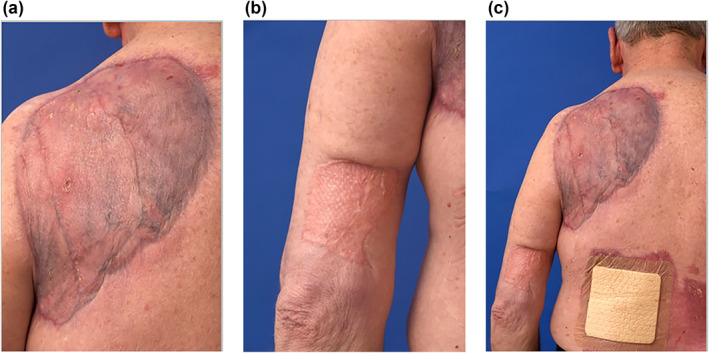
(a) A 4 months after his GBCC resection and reconstruction, our patient's skin grafts had entirely matured. (b) His elbow melanoma site was also reconstructed with a skin graft. (c) There was a small area of breakdown of his donor site (left lumbar back – third photograph, covered with dressing), but otherwise his skin is well‐healed

Adjuvant radiotherapy for the GBCC was discussed with our patient but, due to resection margins, both deep and radial, being ample at more than 20 mm histologically, an active surveillance follow up plan was enacted. His elbow melanoma did receive adjuvant radiotherapy. The patient was followed up at 4 months post excision (Figure 4) and had gained almost 10 kg of weight (due to the removal of the huge catabolic burden of the GBCC), with dietitian guidance, and was grateful to be able to sleep on his back without disturbance. He had moved into a rest home and generally felt safer and far better supported.

## 4 DISCUSSION

Giant BCCs prevail particularly in vulnerable patient cohorts, and they can be a source of humiliation to the patient, further delaying clinical diagnosis and management. The largest BCCs reported in the literature are greater than 30 cm in diameter.^7^ Remarkably, our patient had a clinically treatable cancer. Earlier identification and engagement with healthcare services would have significantly reduced his risk of surgical morbidity. The patient underwent two general anaesthetic procedures and spent a total of 38 days in hospital spread across three admissions. A standard sized BCC can be excised and adequately treated within 30 minutes under local anaesthetic.

Safe surgical margins are yet to be standardized for GBCC – a conservative margin of 2 cm was taken in this particular case, but some data suggests that a ≤1 cm margin is adequate to prevent local recurrence in a median follow up time of 3 years.^1^ GBCC can arise from neglected BCCs in elderly patients and have significant local, regional and systemic complications. His melanoma, due to the patient's decision to not undergo sentinel node biopsy, has an uncertain prognosis. The thickness of his primary melanoma, however, heralds a high metastatic potential, and warrants regular and lengthy surveillance.

Whilst efforts to raise awareness of skin cancers like malignant melanoma is encouraged, we feel that similar efforts should be directed to improving awareness of leaving typically ‘low risk’ BCCs untreated in the community.

## CONFLICT OF INTEREST

None to declare.

## AUTHOR CONTRIBUTIONS

**L. Sun:** Conceptualization; Data curation; Formal analysis; Investigation; Methodology; Project administration; Resources; Software; Validation; Visualization; Writing – original draft; Writing – review & editing. **E. Tan:** Conceptualization; Data curation; Formal analysis; Investigation; Project administration; Writing – original draft.

## Data Availability

The data that support the findings of this study are available from the corresponding author upon reasonable request.
